# Growth differentiation factor 15 as mortality predictor in heart failure patients with non‐reduced ejection fraction

**DOI:** 10.1002/ehf2.12621

**Published:** 2020-06-26

**Authors:** Ana Belen Mendez Fernandez, Andreu Ferrero‐Gregori, Alvaro Garcia‐Osuna, Sonia Mirabet‐Perez, Maria Jose Pirla‐Buxo, Juan Cinca‐Cuscullola, Jordi Ordonez‐Llanos, Eulàlia Roig Minguell

**Affiliations:** ^1^ Department of Cardiology CIBERCV, Hospital Santa Creu i Sant Pau Barcelona Spain; ^2^ Department of Biochemistry IIB‐Sant Pau Barcelona Spain; ^3^ Department of Biochemistry and Molecular Biology Universitat Autònoma Barcelona Spain

**Keywords:** GDF‐15, HFmrEF, HFpEF, Chronic heart failure, Biomarkers

## Abstract

**Aims:**

The prognostic value of biomarkers in patients with heart failure (HF) and mid‐range (HFmrEF) or preserved ejection fraction (HFpEF) has not been widely addressed. The aim of this study was to assess whether the prognostic value of growth differentiation factor 15 (GDF‐15) is superior to that of N‐terminal pro‐brain natriuretic peptide (NT‐proBNP) in patients with HFmrEF or HFpEF.

**Methods and results:**

Heart failure patients with either HFpEF or HFmrEF were included in the study. During their first visit to the HF unit, serum samples were obtained and stored for later assessment of GDF‐15 and NT‐proBNP concentrations. Patients were followed up by the HF unit. The main endpoint was all‐cause mortality. A total of 311 patients, 90 (29%) HFmrEF and 221 (71%) HFpEF, were included. Mean age was 72 ± 13 years, and 136 (44%) were women. No differences were found in GDF‐15 or NT‐proBNP concentrations between both HF groups. During a median follow‐up of 15 months (Q1–Q3: 9–30 months), 98 patients (32%) died, most (71%) of cardiovascular causes. Patients who died had higher median concentrations of GDF‐15 (4085 vs. 2270 ng/L, *P* < 0.0001) and NT‐proBNP (1984 vs. 1095 ng/L, *P* < 0.0001). A Cox multivariable model identified New York Heart Association Functional Class III (*P* = 0.04), systolic blood pressure (*P* = 0.01), left atrial diameter (*P* = 0.03), age >65 years (*P* < 0.0001), and GDF‐15 concentrations (*P* = 0.01) but not NT‐proBNP as independent predictors of all‐cause mortality. The area under the curve was 0.797 for the basic model including NT‐proBNP, and the area under the curve comparing the overall model was 0.819, *P* = 0.016 (DeLong's test). Integrated discrimination improvement index after the inclusion of GDF‐15 in the model with the mortality risk factors was 0.033; that is, the ability to predict death increased by 3.3% (*P* = 0.004). Net reclassification improvement was 0.548 (*P* < 0.001); that is, the capacity to improve the classification of the event (mortality) was 54.8%. GDF‐15 concentrations were divided in tertiles (<1625, 1625–4330, and >4330 ng/L), and survival curves were evaluated using the Kaplan–Meier technique. Patients in the highest tertile had the poorest 5 year survival, at 16%, whereas the lowest tertile had the best survival, of 78% (*P* < 0.001).

**Conclusions:**

Growth differentiation factor 15 was superior to NT‐proBNP for assessing prognosis in patients with HFpEF and HFmrEF. GDF‐15 emerges as a strong, independent biomarker for identifying HFmrEF and HFpEF patients with worse prognosis.

## 1 Introduction

Until recently, heart failure (HF) was classified according left ventricular ejection fraction (LVEF) in HF with reduced or preserved LVEF fraction, although a ‘grey zone’ existed between both HF phenotypes.^1^ The 2016 update of the European Society of Cardiology guidelines defined a new group, HF with mid‐range EF (HFmrEF), as the HF with an LVEF between 40% and 49% and relevant structural heart disease or elevated concentrations of natriuretic peptides.^2^ Thus, HFmrEF shares some characteristics with both HF with preserved EF (HFpEF) and HF with reduced EF (HFrEF) and defines a group with an uncertain prognosis.^3^, ^4^, ^5^, ^6^ HF is a condition resulting from several cardiac processes, such as ischaemia, necrosis, stretch, neurohormonal activation, volume overload, inflammation, and oxidation, and all these processes have their respective biomarkers. As mentioned, HFpEF and HFmrEF patients share some characteristics, including elevated natriuretic peptide concentrations. For this reason, some studies have analysed biomarkers other than natriuretic peptides for improving the prognosis of patients with both HF phenotypes,^7^ but results were inconclusive. The lack of clear differences in the analysed biomarkers and the difficulty in using some of them in daily practice justifies the search for new prognostic biomarkers for both HF phenotypes.

Growth differentiation factor 15 (GDF‐15), also known as macrophage inhibitory cytokine‐1 and non‐steroidal anti‐inflammatory drug‐activated gene‐1, is a member of the transforming growth factor‐β cytokine superfamily with immunosuppressive, anti‐apoptotic, and anti‐inflammatory properties. GDF‐15 is weakly expressed in healthy human tissues, with the exception of the placenta. GDF‐15 expression is up‐regulated by p53, which in turn is a factor responding to daily‐life stressors, such as inflammation, hypoxia, and oxidative stress.^8^ Given the plurality of causes that increase GDF‐15, its circulating concentrations have been related with an increased risk of all‐cause, cardiovascular, and non‐cardiovascular mortality, cardiovascular events such as myocardial infarction, HF hospitalization or stroke, and major bleeding in atrial fibrillation in community studies and in patients with stable or acute coronary disease..^9^ However, GDF‐15 concentrations have been measured in these studies using different methods, the designs of which promoted a heterogeneity in GDF‐15 concentrations that was found to be associated with adverse outcomes. Recently, a fully automatic electrochemiluminescence immunoassay has been made available for clinical use, which will facilitate the measurement of large number of samples with better analytical precision than associated with previous methods.

The aim of our study was to assess the potential of GDF‐15, measured by a fully automated immunoassay, in predicting all‐cause mortality in a large group of HFmrEF and HFpEF patients.

## 2 Methods

Patients meeting HF diagnosis criteria according the European Society of Cardiology guidelines,^2^ referred to our HF unit (HFU) between 2010 and 2015, were included in the study. Patients were referred after an episode of decompensated HF, either after an emergency room visit for dyspnoea or hospital admission for HF; in both cases, HF was the final diagnosis, and all patients were treated with diuretics. All patients underwent echocardiography and were classified according to EF as HFrEF, HFmrEF, or HFpEF. Patients with reduced EF were excluded from the study. Of all patients included, 276 patients had elevated natriuretic peptides [N‐terminal pro‐brain natriuretic peptide (NT‐proBNP) >300 ng/L], while the remaining 35 patients, despite lower concentrations of NT‐proBNP, had relevant structural heart disease (nine had left ventricular hypertrophy, 17 left atrial enlargement, and four implanted pacemaker, and five were in atrial fibrillation). One hundred and fourteen patients had hypertensive cardiomyopathy, 77 ischaemic heart disease, 25 hypertrophic cardiomyopathy, and 83 valve disease. Other miscellaneous aetiologies were three patients with previous cancer treatment, two with non‐compaction cardiomyopathy, and five with atrial fibrillation, all of whom had mild left ventricular dysfunction. The remaining two patients had constrictive pericarditis.

During the first visit to the HFU, signs and symptoms were registered and blood was drawn. Serum samples were obtained after centrifugation and stored at −80 °C until analysis. NT‐proBNP and GDF‐15 were measured by electrochemiluminescence immunoassays on a Cobas e601 platform (Roche Diagnostics, Basel, Switzerland). The measurement ranges for NT‐proBNP and GDF‐15 were 5–35 000 and 400–20 000 ng/L, and precision was ≤3.5% and ≤4.9%, respectively, according to the manufacturer. Values reaching the top of the concentration ranges were equivalent to the maximum detectable value (i.e. 35 000 ng/L for NT‐proBNP and 20 000 ng/L for GDF‐15). Patients were followed in the HFU, and all‐cause mortality was registered. The study was conducted in accordance with the principles outlined in the Declaration of Helsinki. All participants received information about the study and signed informed consent. The study was approved by the centre's ethics committee.

### 2.1 Statistical analyses

Categorical variables were expressed as frequencies and percentages. Continuous variables were expressed as mean ± standard deviation or median (Q1–Q3, first and third quartiles), according to their Gaussian or non‐Gaussian distribution. The statistical differences between groups were compared using the *χ* ^2^ test for categorical variables and the Student's *t*‐test or the Mann–Whitney *U* test according to the Gaussian or non‐Gaussian distribution of the continuous variables. Survival analysis was performed using proportional hazards regression models dividing GDF‐15 into tertiles. The proportionality of the risks was tested using Schoenfeld residuals. The survival curves were also evaluated with the Kaplan–Meier technique for the values of GDF‐15 (analysed by tertiles), and the differences in survival were tested with the log‐rank test. Several variables with known power or power determined in the univariate analysis to identify outcomes were entered in the multivariate analysis. Variables were age, HF aetiology, New York Heart Association (NYHA) functional class, systolic blood pressure, atrial fibrillation, left atrial diameter, estimated glomerular filtration rate, haemoglobin, NT‐proBNP, and GDF‐15 concentrations. A backward elimination method was then used to identify independent predictors of all‐cause mortality. To evaluate the potential value of measured biomarkers in a prediction model of mortality risk, patients with HFmrEF and HFpEF were considered together, given the similarities found between both groups. The potential value of measured biomarkers in a prediction model of mortality risk, which included the independent predictors of mortality found in our cohort, as a base risk multivariable model, in addition to NT‐proBNP, was evaluated by several methods. The Grønnesby and Borgan goodness‐of‐fit test was used to determine if the proposed model fitted the observed outcome with the expected calibration capacity of the model. The concordance index (C‐statistic) checked the discrimination capacity of the model. The improvement in the discrimination capacity of the model including the biomarkers was assessed by the integrated discrimination improvement index, which evaluated the changes in the prediction probabilities of mortality estimated. The net reclassification improvement, category‐free version, was used to test changes in the prediction of estimated mortality implying a change from one category to another.^10^ Finally, the receiver operating characteristic curve of global mortality was calculated, comparing GDF‐15 and NT‐proBNP. A value of *P* < 0.05 was considered statistically significant. Statistical software SPSS Version 24.0 (IBM Corp., Released 2016, IBM SPSS Statistics for Windows, Version 24.0, Armonk, NY) and R Version 3.3.2 (R Foundation for Statistical Computing, Vienna, Austria) were used to perform all statistical analyses.

## 3 Results

**Figure 1:**
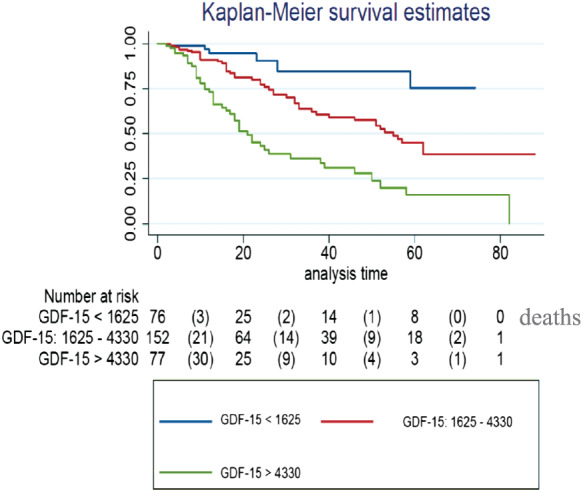
Kaplan–Meier curves showing cumulative survival according to tertiles of growth differentiation factor 15 (GDF‐15) concentrations.

Three hundred and eleven patients were included in the study, of whom 221 had HFpEF and the remaining 90 HFmrEF; their main characteristics are shown in *Table* 1. Patients with HFmrEF were younger and had lower left ventricular EF, more dilated left ventricles, and a higher incidence of ischaemic heart disease than patients with preserved EF. Of note, concentrations of GDF‐15 [2748 ng/L (HFmrEF) vs. 2822 ng/L (HFpEF)] and NT‐proBNP [1362 ng/L (HFmrEF) vs. 1315 ng/L (HFpEF)] did not differ statistically between both groups of patients (*Table* 1). During follow‐up (median 15 months, Q1–Q3: 9–30 months), 98 (32%) patients died (71% of cardiovascular causes); the main cause of death was refractory HF (*Table* 2). *Table* 3 shows the comparison of clinical characteristics of patients who died during follow‐up vs. survivors. Patients who died were older and had lower systolic blood pressure, a higher incidence of atrial fibrillation, worse NYHA functional class, and lower eGFR and haemoglobin than those who survived. Furthermore, they had higher GDF‐15 and NT‐proBNP concentrations than patients with an uneventful follow‐up: GDF‐15: 4085 (inter‐quartile range: 2554–6651) ng/L vs. 2254 (inter‐quartile range: 1389–3562) ng/L (±2674 ng/L), *P* < 0.0001, and NT‐proBNP: 3613 ng/L (±5629 ng/L) vs. 1835 ng/L (±3449 ng/L) expressed as median and standard deviation, *P* < 0.0001. Multivariable analyses identified NYHA Functional Class III (*P* = 0.04), systolic blood pressure (*P* = 0.01), left atrial diameter (*P* = 0.03), age >65 years (*P* < 0.0001), and GDF‐15 concentrations (*P* = 0.01) but not NT‐proBNP as independent predictors of all‐cause mortality (*Table* 4). The area under the curve was 0.797 for the basic model including NT‐proBNP, and the area under the curve comparing the whole model was 0.819, *P* = 0.016 (DeLong's test). Integrated discrimination improvement index after the inclusion of GDF‐15 in the model with the mortality risk factors was 0.033; that is, the ability to predict death increased by 3.3% (*P* = 0.004). Net reclassification improvement was 0.548 (*P* < 0.001); that is, the capacity to improve the classification of the event (mortality) was 54.8%. GDF‐15 concentrations were divided in tertiles (<1625, 1625–4330, and >4330 ng/L) and the respective survival compared by Kaplan–Meier survival curves. Statistically significant differences in 5 year survival were found among the three groups. Patients in the highest tertile had the poorest 5 year survival, at 16%, whereas in the lower tertile, survival was 78% (*P* < 0.001); survival among patients in the intermediate tertile (39%) differed statistically from the lower (*P* = 0.006) and higher (*P* < 0.001) tertiles (*Figure* *1*).

**Table 1:** Main characteristics of all patients and according to left ventricular ejection fraction: mid‐range (40–49%) and preserved (≥50%)

	All	HFmrEF	HFpEF	*P*‐value
	*n* = 311	*n* = 90	*n* = 221	
Age (years)	72 ± 13	67 ± 14	73 ± 12	<0.001
Gender (male)	175 (56%)	66 (73%)	109 (49%)	<0.001
Aetiology				<0.001
Hypertensive cardiomyopathy	114 (37%)	25 (28%)	89 (40%)	0.038
Ischaemic heart disease	77 (25%)	38 (42%)	39 (18%)	<0.001
HCM	25 (8%)	4 (4%)	21 (10%)	0.100
Valve disease	83 (27%)	18 (20%)	65 (29%)	0.089
Miscellaneous	12 (4%)	5 (6%)	7 (3%)	0.339
Hypertension	240 (77%)	65 (72%)	175 (79%)	0.185
Diabetes mellitus	114 (37%)	32 (36%)	82 (37%)	0.797
Dyslipidaemia	150 (48%)	44 (49%)	106 (48%)	0.882
Atrial fibrillation	152 (49%)	34 (38%)	118 (53%)	0.01
Weight (kg)	77 ± 17	78 ± 15	76 ± 17	0.439
BMI (kg/m^2^)	29 ± 5	28 ± 5	29 ± 6	0.175
Blood pressure (mmHg)				
Systolic	131 ± 21	132 ± 20	127 ± 20	0.03
Diastolic	75 ± 11	75 ± 11	75 ± 10	0.818
NYHA‐FC				
II	188 (60%)	61 (68%)	127 (58%)	0.241
III	123 (39%)	29 (32%)	94 (43%)	
eGFR (mL/min/1.73 m^2^)	59 ± 24	62 ± 25	58 ± 23	0.247
Haemoglobin (g/L)	127 ± 19	128 ± 21	127 ± 18	0.515
NT‐proBNP (ng/L)	1346	1362	1315	0.844
(median Q1–Q3)	(542–2651)	(658–2968)	(536–2632)	
GDF‐15 (ng/L)	2822	2748	2822	0.520
(median Q1–Q3)	(1631–4378)	(1218–5253)	(1695–4176)	
Echocardiography				
LVEF (%)	58 ± 12	44 ± 3	64 ± 9	<0.001
LVEDD (mm)	50 ± 8	55 ± 7	47 ± 7	<0.001
IVST (mm)	13 ± 3	12 ± 3	14 ± 4	0.01
LAD (mm)	50 ± 10	49 ± 8	51 ± 9	0.104
MR (Grade 3–4)	40 (13%)	10 (11%)	30 (14%)	0.564
msPAP ≥ 40 mmHg	120 (39%)	27 (43%)	93 (52%)	0.223
msPAP (mmHg)	40 ± 16	38 ± 16	40 ± 15	0.789
*E*/*A*	1.2 ± 0.7	1.3 ± 0.8	1.2 ± 0.6	0.422
Deceleration time (ms)	221 ± 76	221 ± 84	220 ± 72	0.168
Treatment				
ACEIs/ARBs	229 (74%)	77 (86%)	162 (69%)	0.002
Beta‐blockers	201 (65%)	75 (83%)	126 (57%)	<0.001
Loop diuretics	261 (84%)	68 (76%)	193 (87%)	0.01
MRA	110 (35%)	41 (46%)	69 (31%)	0.01

ACEIs, angiotensin‐converting enzyme inhibitors; ARBs, angiotensin receptor blockers; BMI, body mass index; *E*/*A*, early (*E*) mitral inflow peak/atrial (*A*) filling peak ratio; eGFR, estimated glomerular filtration rate; GDF‐15, growth differentiation factor 15; HCM, hypertrophic cardiomyopathy; HFmrEF, heart failure with mid‐range ejection fraction; HFpEF, heart failure with preserved ejection fraction; IVST, interventricular septum thickness; LAD, left atrial diameter; LVEDD, left ventricular end‐diastolic diameter; LVEF, left ventricular ejection fraction; MR, mitral regurgitation; MRA, mineralocorticoid receptor antagonist; msPAP, mean estimated systolic pulmonary artery pressure; NT‐proBNP, N‐terminal pro‐brain natriuretic peptide; NYHA‐FC, New York Heart Association functional class.

**Table 2:** Causes of death

	*n* (%)
CV death	
Heart failure	59 (60%)
Arrhythmia	6 (6.1%)
AMI	2 (2.0%)
CVA	3 (3.0%)
Non‐CV death	
Neoplasia	7 (7.3%)
Infection	6 (6.1%)
Renal failure	4 (4.0%)
Aortic aneurism	2 (2.4%)
Vascular surgery	2 (2.4%)
Unknown	7 (7.3%)

AMI, acute myocardial infarction; CV, cardiovascular; CVA, cerebrovascular accident.

**Table 3:** Main characteristics of patients grouped according the occurrence of all‐cause mortality

	Alive	Died	*P*‐value
	*n* = 213	*n* = 98	
Gender (male)	119 (44%)	56 (32%)	0.833
Aetiology			
Hypertensive cardiomyopathy	78 (37%)	36 (37%)	0.984
Ischaemic	54 (25%)	23 (23%)	0.721
HCM	19 (9%)	6 (6%)	0.399
Valve disease	54 (25%)	29 (30%)	0.432
Miscellaneous	8 (4%)	4 (4%)	1.000
Blood pressure (mmHg)			
Systolic	134 ± 22	125 ± 19	0.002
Diastolic	76 ± 12	73 ± 11	0.076
Hypertension	158 (74%)	82 (84%)	0.081
Diabetes mellitus	78 (37%)	36 (37%)	1.000
Dyslipidaemia	104 (49%)	46 (47%)	0.807
Atrial fibrillation	88 (41%)	64 (65%)	0.0001
NYHA‐FC			
II	137 (64%)	51 (52%)	0.002
III	76 (36%)	47 (48%)	
eGFR (mL/min/1.73 m^2^)	64 ± 23	49 ± 21	0.0001
Haemoglobin (g/L)	129 ± 18	123 ± 19	0.01
NT‐proBNP (ng/L)	1095	1984	0.0001
(median Q1–Q3)	(145–2188)	(880–4852)	
GDF‐15 (ng/L)	2270	4085	0.0001
(median Q1–Q3)	(1403–3596)	(2554–6756)	
Echocardiography			
LVEF (%)	57 ± 12	60 ± 11	0.02
LVEDD (mm)	50 ± 8	49 ± 8	0.455
IVST (mm)	13 ± 3	14 ± 4	0.626
LAD (mm)	48 ± 8	52 ± 10	0.001
MR (Grade 3–4)	24 (11%)	16 (16%)	0.197
msPAP ≥ 40 mmHg	73 (45%)	47 (60%)	0.03
msPAP (mmHg)	38 ± 15	43 ± 16	0.01
*E*/*A*	1.1 ± 0.7	1.6 ± 0.9	0.01
Deceleration time (ms)	225 ± 76	198 ± 69	0.734
Treatment			
ACEIs/ARBs	160 (75%)	69 (70%)	0.407
Beta‐blockers	149 (70%)	52 (53%)	0.004
Loop diuretics	166 (78%)	95 (97%)	0.0001
MRA	65 (30%)	45 (46%)	0.008

ACEIs, angiotensin‐converting enzyme inhibitors; ARBs, angiotensin receptor blockers; BMI, body mass index; *E*/*A*, early (*E*) mitral inflow peak/atrial (*A*) filling peak ratio; eGFR, estimated glomerular filtration rate; GDF‐15, growth differentiation factor 15; HCM, hypertrophic cardiomyopathy; IVST, interventricular septum thickness; LAD, left atrial diameter; LVEDD, left ventricular end‐diastolic diameter; LVEF, left ventricular ejection fraction; MR, mitral regurgitation; MRA, mineralocorticoid receptor antagonist; msPAP, mean estimated systolic pulmonary artery pressure; NT‐proBNP, N‐terminal pro‐brain natriuretic peptide; NYHA‐FC, New York Heart Association functional class.

**Table 4:** Multivariable analyses for all‐cause mortality

	Hazard ratio	95% confidence interval		Significance
		Lower	Upper	
NYHA‐FC III	1.645	1.013	2.670	0.044
SBP	0.983	0.971	0.996	0.011
LAD	1.020	1.002	1.038	0.031
Age	1.072	1.043	1.102	0.000
GDF‐15 per 100 ng/L	1.008	1.003	1.013	0.01
NT‐proBNP per 100 ng/L	1.000	1.000	1.000	0.328

GDF‐15, growth differentiation factor 15; GDF‐15 per 100 ng/L, risk per each 100 ng/L increase; LAD, left atrial diameter; NT‐proBNP, N‐terminal pro‐brain natriuretic peptide; NT‐proBNP per 100 ng/L, risk per each 100 ng/L increase; NYHA‐FC, New York Heart Association functional class; SBP, systolic blood pressure.

Grønnesby and Borgan test shows a good model calibration: *P* = 0.769.

## 4 Discussion

Our results suggest that GDF‐15 is a useful biomarker for identifying HF patients with non‐reduced EF associated with worse prognosis. Moreover, reduced functional capacity, low systolic blood pressure, higher left atrial diameter, older age, and higher GDF‐15 concentration were the clinical variables identified as predictors of mortality. Thus, GDF‐15 was the only biomarker with prognostic value identified in these patients. Natriuretic peptides are recommended in clinical practice for assessing the diagnosis of HF independently of LVEF.^2^, ^10^ Moreover, they are also recommended for the assessment of prognosis in patients with HFrEF. However, their predictive value in patients with HFmrEF or HFpEF is less clear,^11^ and several factors can account for this controversy. In the case of NT‐proBNP, different cut‐off values according to patient age have been suggested, but this could complicate individual risk evaluation and stratification. Furthermore, previous studies used different LVEF values to classify patients with low or preserved EF. In fact, HFmrEF is a very heterogeneous group of patients that can include patients with recovered EF after previous reduced ventricular function and patients with persistent mild deterioration. In a recent publication of the Swedish HF registry,^12^ which divided patients according to the new HF classification, the prognostic value of elevated NT‐proBNP was maintained independently of EF. Similarly, increased BNP concentrations, although lower in patients with HFpEF than in patients with HFrEF, were also associated with worse prognosis.^13^ Despite these results, new biomarkers are now available,^14^ which can improve prognosis prediction in these patients. Previous studies have shown that GDF‐15 is elevated in HFpEF or HFmrEF patients to a similar degree as in HFrEF patients.^15^ Increased GDF‐15 has been shown to be independently associated with exercise capacity impairment and poor quality of life,^16^ and its accuracy has been at least as good as that of NT‐proBNP, all suggesting that the combination of both markers could improve diagnostic power. GDF‐15 is secreted from the myocardium in response to various stimuli such as ischaemia, wall stress, or pressure overload.^17^ It is also secreted from fibroblasts in response to stress stimuli.^18^ Thus, although the mechanisms are still unclear, both cardiomyocytes and heart fibroblasts could be the source of GDF‐15 in patients with HF. Because cardiac hypertrophy and interstitial fibrosis are common pathological features of HFpEF and HFmrEF, the prognostic value of GDF‐15 found in these patients is not entirely surprising. Furthermore, GDF‐15 is also a marker of inflammation and metabolic syndrome frequently seen in patients with HF. The association of metabolic co‐morbidities such as obesity and diabetes can induce coronary microvascular inflammation. These metabolic changes can increase cardiomyocyte stiffness because of limited availability of nitric oxide and induce fibrosis because of myocardial infiltration by activated macrophages. Experimental studies have also demonstrated that GDF‐15 null mice show exacerbated cardiac hypertrophy in response to pressure overload, suggesting that GDF‐15 has antihypertensive properties.^17^ However, no significant correlation between serum GDF‐15 level and left ventricular mass index has been found in a recent study.^19^ In a recent study of 70 patients with HFpEF, elevated GDF‐15 was associated with higher BNP values and identified as an independent predictor of adverse cardiovascular outcomes.^20^ Similarly to our results, in the study by Lok *et al*.^21^ that included patients with advanced HF, GDF‐15 was also superior to NT‐proBNP in assessing prognosis. However, in that study, patients were selected according to NYHA Functional Class III–IV, but no information was provided on their ventricular function status. Unlike other studies, we did not find any prognostic power for NT‐proBNP in patients with HFpEF and HFmrEF, perhaps because the clinical variables identified as predictors of worse prognosis are variables tightly correlated with NT‐proBNP concentrations, and consequently, the biomarker may have lost statistical significance for predicting events. However, because GDF‐15 is a marker of fibrosis and inflammation, it may play a more important role in the pathophysiology of HFpEF and HFmrEF and consequently in the prognosis of these conditions. Recent studies have also suggested that a model of several biomarkers may provide higher prognostic information in HF patients,^22^ but these models are expensive and not widely available. Thus, GDF‐15 concentrations add prognostic value to clinical information and emerge as a new biomarker that may help to assess prognosis in patients with HF. In our study, we measured GDF‐15 with a fully automatic immunoassay that showed remarkably low analytical imprecision. In previous studies, GDF‐15 was mainly measured with radioimmunometric^23^ or enzyme‐linked immunosorbent assays^20^; these analytical approaches usually show higher imprecision than fully automated methods. Indeed, the method we used is developed on an immunoassay platform available in many clinical laboratories, thus facilitating the transferability of results among different studies. In conclusion, GDF‐15 emerged as an independent predictor of all‐cause mortality in patients with LVEF > 40%. Furthermore, in our cohort, GDF‐15 was superior to NT‐proBNP in the prognostic assessment of patients with HFpEF and HFmrEF.

### 4.1 Limitations

The low number of patients recruited from a single centre may be the main limitation of the study. However, it is one of the largest studies to report the use of GDF‐15 for prognostic assessment, and internal validation was applied. Despite the fact that patients with preserved or mid‐range EF were analysed together, there were no significant differences in biomarker concentrations between them. Furthermore, the cause of HF in our study was heterogeneous, so extreme care should be taken when interpreting these results.

### 4.2 Clinical perspectives

New biomarkers are needed to identify high‐risk patients with HFmrEF and HFpEF to improve their prognosis. Higher GDF‐15 values are associated with higher mortality in patients with HFpEF and HFmrEF. However, more studies are necessary before the implementation of GDF‐15 in clinical practice can be recommended. Our preliminary results should be corroborated in larger cohorts.

## Conflict of interest

None declared.

## Funding

This study was supported by Fundació d'Investigació Sant Pau (G‐60136934).
